# Sensor histidine kinases *kdpD* and *aauS* regulate biofilm and virulence in *Pseudomonas aeruginosa* PA14

**DOI:** 10.3389/fcimb.2023.1270667

**Published:** 2023-10-10

**Authors:** Maria Sultan, Rekha Arya, Akhilesh Kumar Chaurasia, Kyeong Kyu Kim

**Affiliations:** ^1^ Department of Precision Medicine, Graduate School of Basic Medical Science, Institute for Antimicrobial Resistance Research and Therapeutics, Sungkyunkwan University School of Medicine, Suwon, Republic of Korea; ^2^ Department of Orthopedic Surgery, University of Pittsburgh School of Medicine, Pittsburgh, PA, United States

**Keywords:** *Pseudomonas aeruginosa*, two-component system (TCS), sensor histidine kinase, KdpD, AauS, quorum sensing (QS), virulence

## Abstract

*Pseudomonas aeruginosa* is a multidrug-resistant opportunistic human pathogen that utilizes two-component systems (TCSs) to sense pathophysiological signals and coordinate virulence. *P. aeruginosa* contains 64 sensor histidine kinases (HKs) and 72 response regulators (RRs) that play important roles in metabolism, bacterial physiology, and virulence. However, the role of some TCSs in virulence remains uncharacterized. In this study, we evaluated the virulence potential of some uncharacterized sensor HK and RR knockouts in *P. aeruginosa* using a *Galleria mellonella* infection model. Furthermore, we demonstrated that KdpD and AauS HKs regulate virulence by affecting *P. aeruginosa* biofilm formation and motility. Both Δ*kdpD* and Δ*aauS* showed reduced biofilm and motility which were confirmed by restored phenotypes upon complementation. Moreover, Δ*kdpD* and Δ*aauS* exhibited increased survival of HeLa cells and *G. mellonella* during *in vivo* infection. Altered expression of the transcriptional regulators *anR* and *lasR*, along with the virulence genes *lasA, pelA, cupA, pqsA, pqsB, pqsC*, and *pqsD* in the mutant strains elucidated the mechanism by which Δ*kdpD* and Δ*aauS* affect virulence. These findings confirm that *kdpD* and *aauS* play important roles in *P. aeruginosa* pathogenesis by regulating biofilm formation and motility.

## 1 Introduction

Two-component systems (TCSs) play essential roles in the adaptation and survival of bacteria in various stressful environments, including pathophysiological conditions (Mitrophanov and Groisman, 2008; De Bentzmann and Plésiat, 2011). A TCS generally comprises a sensor histidine kinase (HK) and its cognate response regulator (RR). During pathogenesis, the HK responsible for sensing pathophysiological stimuli is phosphorylated and subsequently transfers or phosphorelays the signal to its cognate RR to activate the expression of virulence genes under its control (Stock et al., 2000). Bacterial virulence depends on virulence determinants, including biofilm formation, motility, lipopolysaccharides, capsules, alginate, pyoverdine, and pyocyanin. It also depends on the secretion of toxins, such as exotoxin A, phospholipases, and proteases, which play vital roles in disease progression caused by *P. aeruginosa* (Leitão, 2020; Jurado-Martín et al., 2021).

*P. aeruginosa* is a Gram-negative, multidrug-resistant, opportunistic bacterium responsible for severe community and nosocomial infections in immunocompromised patients (De Bentzmann and Plésiat, 2011; Wu et al., 2014; Gale et al., 2015; Moradali et al., 2017). *P. aeruginosa* causes approximately 7.1–7.3% of healthcare-associated infections (Magill et al., 2014; Weiner et al., 2016), and is the main cause of mortality and morbidity in patients with cystic fibrosis (CF) (Mogayzel et al., 2014). In a major multinational observational study of the point prevalence of infections in intensive care units, *P. aeruginosa* was reported to be responsible for 16.2% of patients’ infections and 23% of all ICU-acquired infections (Vincent et al., 2020). It is a life-threatening bacterium that was included in the priority pathogen list in 2017 (World Health Organization, 2017).

The *P. aeruginosa* genome contains many TCSs, including 64 sensor HKs and 72 RRs (Francis et al., 2017). These TCS-controlling networks are promising targets against antimicrobial resistance (AMR), pathogenesis, and biofilm (Francis et al., 2017; Tierney and Rather, 2019).

Biofilms are organized bacterial communities that act as buffers against external environmental stress (Yin et al., 2019). They may form colonies and persist long-term in patients by escaping the host immune system (Kostakioti et al., 2013). Moreover, extremely structured biofilms have been detected in patients with chronic lung and wound infections (Römling and Balsalobre, 2012). *P. aeruginosa* is a major biofilm-forming bacterium and the primary cause of 65–80% of nosocomial infections (Li et al., 2023). Motility (swarming, swimming, and twitching) is another important virulence factor in pathogenesis, as it is crucial for mobilization, colonization in multiple environments, adherence, and biofilm formation (O'Toole and Kolter, 1998). Motility is involved in biofilm formation in many model bacterial pathogens (Korber et al., 1994; Pratt and Kolter, 1998).

Previous studies have demonstrated the roles of many TCSs in virulence (Francis et al., 2017; Sultan et al., 2021). The TCS GacS/GacA and HKs RetS and LadS act as switches between planktonic and biofilm lifestyles in *P. aeruginosa* (Mikkelsen et al., 2011; Chambonnier et al., 2016). BfiS/BfiR, MifS/MifR, and BfmS/BfmR TCSs contribute to biofilm development in a stage-specific manner and cause irreversible attachment, microcolony formation, and maturation, respectively (Petrova and Sauer, 2009). FleS/FleR, another TCS, regulates the expression of flagellar biosynthetic genes and alters swarming motility (Dasgupta et al., 2003). Similarly, CarS/CarR is a well-recognized calcium homeostasis TCS that regulates swarming motility and calcium-induced virulence factors (Guragain et al., 2016). However, the roles of some TCSs in virulence and biofilm formation in *P. aeruginosa* PA14 warrant further elucidation. Therefore, it is necessary to identify uncharacterized TCSs and establish their roles in virulence and biofilm formation to better understand *P. aeruginosa* pathogenesis.

The functional roles of *kdpD, aauS, nasT*, and *cheB* in *P. aeruginosa* virulence are not well characterized (Francis et al., 2017). Therefore, we monitored their potential for infection in a *G. mellonella* infection model using isogenic knockout strains to investigate their role in virulence. Based on the initial test, *kdpD* and *aauS* played a more substantial role in infection than *nasT* and *cheB.* These two HKs are involved in bacterial physiology. *kdpD* is an essential gene for potassium (K^+^) transport (Freeman et al., 2013), whereas *aauS* is involved in acidic amino acid utilization (Sonawane and Singh, 2006). However, their roles in *P. aeruginosa* virulence have not been well characterized. Recently, Badal et al. (2020) developed an *in vitro* approach to screen the role of 112 TCS of *P. aeruginosa* in biofilm on endotracheal tubes. Out of 112, 56 TCS were found to be involved in biofilm. Of these, 18 new TCSs including AauS and KdpD were identified to contribute in biofilm (Badal et al., 2020). However, the current study elucidated the role of KdpD and AauS in motility; and virulence by using HeLa cell line, and *G. mellonella* infection model. We further demonstrated the involvement of KdpD and AauS in biofilm using functional genomics and gene expression analyses of relevant genetic cascades to elucidate the mechanism by which *kdpD* and *aauS* affect the biofilm formation, motility, and pathogenesis of *P. aeruginosa* PA14.

## 2 Materials and methods

### 2.1 Growth and culture conditions of bacterial strains

Non-redundant transposon mutant libraries containing Δ*nasT*, Δ*cheB*, Δ*kdpD*, and Δ*aauS* that were developed using PA14 (PA14NR) (Liberati et al., 2006) were obtained from Harvard Medical School. All strains were grown on cetrimide agar (CA) (Sigma-Aldrich, St. Louis, MO, USA) plates for 16–18 h at 37 °C. Bacterial cultures were grown in Luria-Bertani (LB) medium (Becton Dickinson, Franklin Lakes, NJ, USA) under orbital shaking culture conditions at 200 rpm. Gentamicin (15 μg/mL) was used to grow the mutants, whereas 50 µg/mL carbenicillin (Sigma-Aldrich) was used to grow the strains harboring empty pUCP18 or its derivative plasmids. The mutant complementation strains were grown in the presence of 15 μg/mL gentamicin and 50 μg/mL carbenicillin. Bacterial growth was monitored by measuring the optical density at 600 nm [OD_600nm_ = 1.0 = 1.0 × 10^9^ colony-forming units (CFU/mL)] using a Gen 5 BioTek microplate reader (BioTek, Winooski, VT, USA). Bacterial cells were harvested using 4000 rpm (2701 ×*g*) centrifugation at 4 °C. They were washed once using phosphate buffered saline (PBS, pH 7.2) in all experiments, unless otherwise stated. The strains and plasmids, as well as primers used in the study are listed in **Supplementary Tables S1, S2**, respectively.

### 2.2 Complementation of Δ*kdpD* and Δ*aauS* mutant strains

To complement the transposon mutants Δ*kdpD* and Δ*aauS*, the corresponding genes (*kdpD* and *aauS*) were PCR-amplified from *P. aeruginosa* PA14 genomic DNA using specific primers (**Table S2**). The column purified PCR-amplified DNA fragments, and the pUCP18 vector were restriction endonuclease digested with EcoRI and HindIII. The EcoRI/HindIII-digested *kdpD* and *aauS* genes and pUCP18 vector DNA were resolved on an agarose gel. This was followed by gel elution using a Cosmo Genentech gel elution kit (Seoul, Korea). The gel-eluted *kdpD* and *aauS* were cloned into EcoRI/HindIII sites of the pUCP18 vector to yield the recombinant plasmids pUCP18*kdpD* and pUCP18*aauS*, respectively. These plasmids were transformed into *Escherichia coli* DH5α, and clones were selected on LB agar plates containing 50 μg/mL carbenicillin. The plasmids were isolated using a Cosmo Genentech plasmid isolation kit and DNA was sequenced to verify the accuracy of the nucleotide sequences of the genes in pUCP18*kdpD* and pUCP18*aauS*. The empty control plasmid, pUCP18, was transformed into wild-type (WT), Δ*kdpD* and Δ*aauS* of *P. aeruginosa* PA14. The WT strain possessing pUCP18 (WT::pUCP18) was selected on LB agar plates containing 50 μg/mL carbenicillin. Furthermore, the mutant strains (Δ*kdpD* and Δ*aauS*) possessing the empty vector (Δ*kdpD*::pUCP18 and Δ*aauS*::pUCP18) were selected on LB-agar plates containing 15 μg/mL gentamicin and 50 μg/mL carbenicillin. To obtain the knockout-complemented strains (Δ*kdpD*::pUCP18*kdpD* and Δ*aauS*::pUCP18*aauS)*, the recombinant plasmids pUCP18*kdpD and* pUCP18*aauS* were transformed into Δ*kdpD* and Δ*aauS* knockouts and selected on LB plates containing 50 μg/mL carbenicillin and 15 μg/mL gentamicin.

### 2.3 Quantification of biofilm formation using a crystal violet assay

Biofilm quantification through crystal violet (CV) staining was performed as previously described (Chen et al., 2018). Briefly, 16 h cultures of WT, mutant, and mutant-complemented strains were grown in Muller Hinton broth (MHB) (Becton Dickinson) at 37 °C and diluted to OD_600_ = 1 × 10^8^ CFU/mL in fresh MHB media. An aliquot of 100 μL bacterial suspension was added to a flat-bottom 96-well polystyrene microtiter plate. After 24 or 48 h incubation at 37 °C, unattached cells were removed using two washes of PBS (pH 7.2). Next, 100 μL of 99% methanol was added to each well for 15 min, followed by methanol removal and drying of the plates at room temperature (25±1 °C) to perform biofilm fixation. Then, 100 μL of 0.04% CV was added for 20 min to stain the biofilm. Samples were washed with sterile PBS three consecutive times to remove additional colorants. Finally, 33% acetic acid was used to solubilize the biofilm-bound CV. Absorbance was measured at 630 nm using a Gen5 BioTek microplate reader (BioTek Instruments, Inc., Agilent Technologies, CA, USA). *pqsA* is an important biofilm producing gene so, Δ*pqsA* used as a negative control in the current experiment.

### 2.4 Confocal laser scanning microscopy

Bacteria cultures were grown in MHB at 37 °C for 16–18 h and diluted to OD_600nm_ = 1 (1×10^8^ CFU/mL) in fresh MHB media. Then, 500 μL bacterial suspension was added into an 8-well chambered cover glass (Lab-Tek II 1.5 Borosilicate glass, Sigma-Aldrich) and incubated at 37 °C under static culture conditions. The culture medium was changed after 24 h to remove non-attached bacterial cells. After 24 or 48 h of incubation, the biofilms were gently washed with saline [0.85% (w/v) sodium chloride (NaCl)]. Final concentrations of 30 μM propidium iodide (PI), and 5 μM SYTO9 (Sigma-Aldrich) were used for biofilm staining (Chaurasia et al., 2016). The chambered cover glass was incubated in the dark at room temperature for 20 min. The stained biofilms were washed with PBS prior to confocal microscopy. Biofilms were examined using confocal laser scanning microscopy (CLSM, Zeiss, Oberkochen, Germany) at 20× or 63× magnifications. Image stacks were collected from 20 random points on each biofilm to obtain accurate mean values for biofilm thickness measurement.

### 2.5 Swarming motility assay

Swarming motility was assessed as previously described, with slight modifications (Ha et al., 2014a). M8-supplemented swarming medium was used to prepare the swarm plates. Briefly, 6 g agar was added to 800 mL water (final concentration = 0.6%) and autoclaved to obtain a sterile, homogeneous agar suspension. A 5× M8 solution was prepared using 30 g Na_2_HPO_4_, 15 g KH_2_PO_4_, and 2.5 g NaCl in water by adjusting the final volume to 1 L, autoclaving, and finally adding a 5× M8 solution (200 mL) to the melted agar. Next, 20% glucose (final concentration = 0.2%), 20% casamino acids (final concentration = 0.5%), and 1 M MgSO_4_ (final concentration = 1 mM) were added to molten agar. The agar medium was mixed gently and cooled before being poured into Petri dishes (~25 mL/plate) and solidified at room temperature. The center of each plate was inoculated with 2.5 μL of OD_600nm_ 1 equivalent overnight bacterial culture. Plates were incubated upright at 37 °C for 16–24 h and observed for the swarming phenotype. The sensor kinase *fleS* of the *fleSR* TCS was used as a negative control in the current experiment, as Δ*fleS* alters swarming motility in *P. aeruginosa* PA14 (Kollaran et al., 2019).

### 2.6 Swimming motility assay

Swimming motility was assessed using 0.3% LB agar as previously described, with slight modifications (Déziel et al., 2001). First, 25 mL media plates were solidified at room temperature for 1.5 h. The cell number was adjusted based on the OD_600nm_ value. Then, 100 μL of OD_600nm_ 1 bacterial culture was transferred into a 1.5 mL microcentrifuge tube. To avoid the production of swarming and twitching phenotypes, a sterile toothpick was dipped into the OD_600nm_ 1 equivalent culture and stabilized in the middle of the agar layer without touching the top or bottom of the Petri plate. The plates were incubated upright for 20 h at 30 °C. The phenotype was observed, and the diameters of the swim zones were measured. The hook-associated gene, *flgK*, is essential for flagellar assembly. Additionally, its mutant showed reduced swimming motility (Ha et al., 2014b). Therefore, in the current experiment Δ*flgK* was used as a negative control for the no-swimming phenotypes.

### 2.7 Twitching motility assays

Twitching motility was tested as previously described (Déziel et al., 2001). Twitching motility was assessed on LB plates containing 1% (w/v) agar. A sterile toothpick was dipped into the OD_600nm_ 1 equivalent bacterial culture. The toothpick touched the bottom of the Petri plate to move into the interstitial space between the basal surfaces of the agar. The plates were subsequently incubated at 37 °C for 48 h. The agar was gently removed from the Petri plate using a spatula to measure the twitch zone. The twitch zone was stained with 2 mL 0.1% (w/v) CV in water for 10 min. The CV was removed, and the plates were washed with water and allowed to air-dry at room temperature. The twitch zone diameters were measured and recorded. As the role of the TCS PilS/PilR in the regulation of type IV pilus expression has been demonstrated (Kilmury and Burrows, 2018a), we used Δ*pilR* as a negative control.

### 2.8 Quantitative reverse-transcription polymerase chain reaction analysis

The WT, mutant, and complementation strains were grown in M9 minimal medium for 10 h at 37 °C (Egli, 2015). For identification of biofilm genes bacteria was grown in 8-well chambered cover glass for 10, 24 and 48 h. Planktonic cells were discarded, and biofilm population was used for RNA isolation (Bisht et al., 2021). Total RNA was isolated using the Qiagen RNeasy kit (Hilden, Germany) according to the manufacturer’s protocol. Total RNA (1 µg) was treated with RNase-free DNase I (1 U) (amplification grade; Sigma-Aldrich) at room temperature for 15 min. The DNase I reaction was stopped using stop-buffer followed by inactivation of DNase at 70 °C for 10 min. To prepare cDNA from the isolated DNA-free mRNA, a random hexamer premix (RNA-to-cDNA EcoDryTM premix; Takara Bio, Kusatsu, Japan) was used. The qRT-PCR was performed using 55 °C annealing temperature and SYBR Green Supermix (Bio-Rad, Hercules, CA, USA). Here, *rho* was used as a housekeeping gene. The expression of *kdpD* and *aauS* was normalized to calculate the relative gene expression analyzed using the 2^−ΔΔCT^ method (Schmittgen and Livak, 2008).

### 2.9 Invasion assay

To examine the roles of *kdpD* and *aauS* in *P. aeruginosa* virulence, we performed invasion assays as previously described (Kim et al., 2019). HeLa cells were grown in Dulbecco’s modified Eagle’s medium (DMEM) with 10% fetal bovine serum (FBS) in a 5% CO_2_ humidified incubator at 37 °C. The cells were seeded in 6-well plates (5.0×10^5^ cells/well) and incubated for 24 h. PBS-washed bacterial cells were placed in invasion medium (DMEM without FBS) for 2 h before infection. Then, WT and mutant strains at a multiplicity of infection *(*MOI*)* of 10 were used to infect HeLa cells for 120 min. Extracellular bacterial cells were killed through 60-min gentamicin (100 µg/mL) treatment. Residual gentamicin was removed by washing the cells twice with PBS. The infected cells were treated with 0.1% Triton-X100 to lyse the HeLa cells and retrieve intracellular bacteria, which were then serially diluted in PBS to plate 100 µL diluted cell suspension on cetrimide agar plates for CFU enumeration.

### 2.10 Live/dead assays

The live/dead assay was performed as previously described (Mittal et al., 2014). Briefly, DMEM with 10% FBS was used to grow HeLa cells in a 5% CO_2_ humidified incubator at 37 °C for 48 h. HeLa cells were seeded into 8-well chamber slides (5.0×10^5^ cells/well) and incubated for 24 h. After two PBS washes, HeLa cells were infected with bacteria at an MOI of 10 for 120 min. Uninfected cells were used as negative (untreated) controls. After incubation, the infected HeLa cells were washed with PBS and a live/dead viability kit was used for staining. Fluorescently stained bacterial cells were observed using CLSM (Zeiss, Oberkochen, Germany) and images were captured.

### 2.11 *G. mellonella* infection model

The overnight grown cultures of *P. aeruginosa* strains were suspended in LB for 6 h. Bacterial cells were collected through 4000 rpm centrifugation (2701 ×g) at 4 °C and washed with PBS (pH 7.2). The number of bacterial cells was maintained by adjusting the optical density (OD_600 nm_ = 1) in PBS. *G. mellonella* larvae were stored for 24 h before infection (Ménard et al., 2021). Then, the weight and length of each worm were measured. Ten waxworms of similar size (200 ± 2 mg, 2 ± 0.2 cm) were chosen for each group, including the carrier (PBS) control. Waxworms were injected with 10 bacterial cells (20 µL) in the last posterior leg using a 0.3 mL syringe (Becton Dickinson) (Desbois and Coote, 2011; Hill et al., 2014; Imdad et al., 2018). Similarly, 20 µL PBS was injected into the worms in the control group. After injection, worms were incubated at 27 °C and observed at different time points. We applied a health scoring index wherein each worm was given a score based on movement, melaninization, cocoon formation, and survival compared to the untreated control group (Champion et al., 2018). The experiment was terminated after 72 h and CFUs were counted using CA plates.

## 3 Results

### 3.1 Functional assessment of TCSs in virulence using the *G. mellonella* infection model

**Figure 1:**
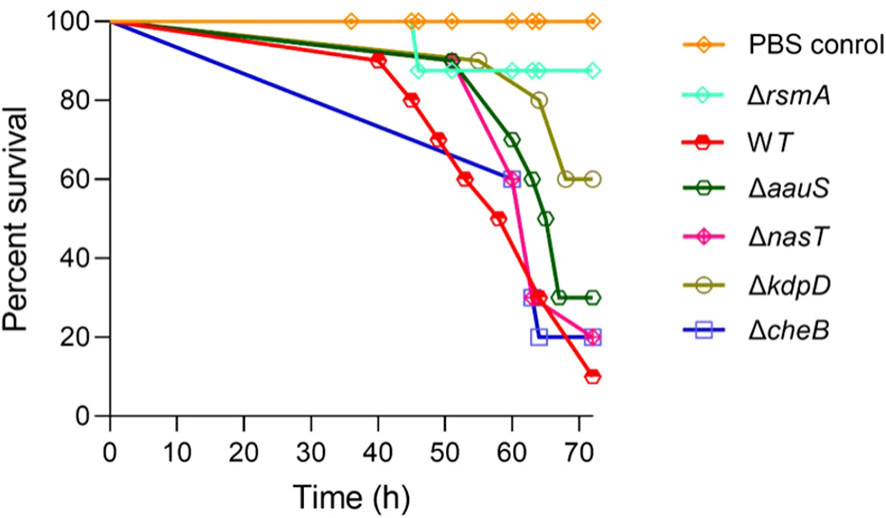
Screening of TCS genes relevant to virulence using a *G. mellonella* larvae infection model. The colony farming units (CFUs) (10 for each strain), namely the WT, Δ*rsmA*, Δ*kdpD*, Δ*aauS*, Δ*nasT,*or Δ*cheB*, were injected into waxworms (*n* =10), followed by incubation at 27 °C to examine their survival up to 72 h post-infection. A delay in mortality was observed for Δ*kdpD* and Δ*aauS*, with 60% and 30% survival, respectively.

The relevance of *kdpD*, *aauS*, *nasT*, and *cheB* in *P. aeruginosa* virulence is yet to be well characterized (Francis et al., 2017). Therefore, we initially tested their roles in virulence using a *G. mellonella* infection model. The hemocytes present in *G. mellonella* larvae share functional similarities with mammalian phagocytic cells (Browne et al., 2013). Therefore, *G. mellonella* larval infection models have been used to identify virulence-related genes in various human pathogens (Jander et al., 2000; Champion et al., 2009; Lebreton et al., 2009; Peleg et al., 2009; Champion et al., 2010). *rsmA*, a virulence gene encoding the carbon storage and a pyocyanin production regulator, was used as a positive control to determine the potential roles of uncharacterized regulators. Mutant strains lacking *rsmA, kdpD*, *aauS*, *nasT*, *cheB*, or the WT were injected into the left posterior legs of *G. mellonella* larvae (n = 10) to assess their roles in the pathogenic potential of *P. aeruginosa* PA14. The results showed only 10% survival in WT strains 72 h post-infection, whereas the Δ*rsmA* showed 90% survival compared to the PBS control (100% survival) (**Figure 1**; **Supplementary Figure S1**). However, a delay in mortality was observed for Δ*kdpD* and Δ*aauS*, with 60% and 30% survival, respectively. The comparative growth profile of Δ*kdpD* and Δ*aauS* knockouts with the WT strain was monitored for physiological fitness to confirm that the difference in their infection potential was not due to their compromised growth proficiency. The growth of the WT, Δ*kdpD*, and Δ*aauS* strains was identical (**Supplementary Figure S2**). Furthermore, the Δ*nasT* and Δ*cheB* strains showed earlier mortality than the WT, with only 20% survival 72 h post-infection. These results demonstrate that *kdpD* and *aauS* contribute to virulence. Thus, we further investigated their relevance to the virulence potential of *P. aeruginosa.*

### 3.2 Roles of *kdpD* and *aauS* in biofilm formation

**Figure 2:**
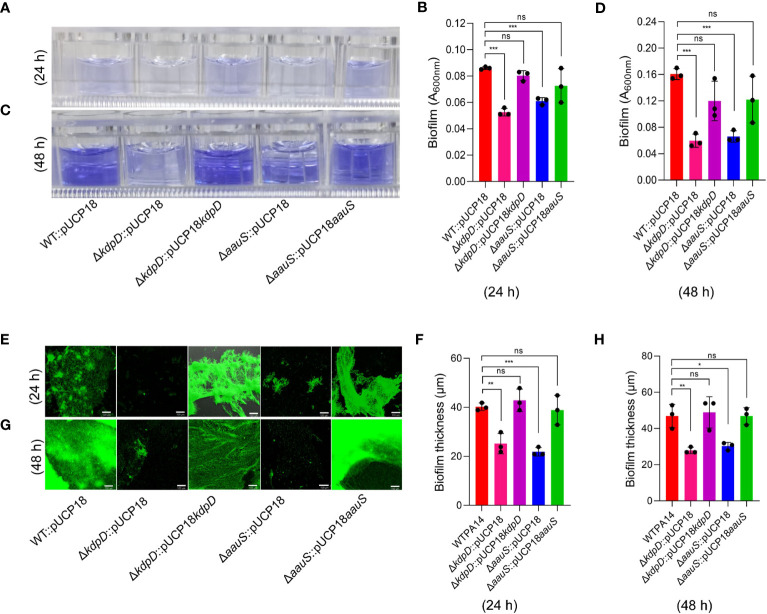
The roles of *kdpD* and *aauS* in biofilm formation. Qualitative and quantitative analysis of biofilm formation by WT::pUCP18, Δ*kdpD*::pUCP18, Δ*aauS*::pUCP18, Δ*kdpD*::pUCP18*kdpD*, and Δ*aauS*::pUCP18*aauS* at 24 and 48 h, performed using 96-well polystyrene plates **(A–D)** and 8-chambered covered glass slides **(E–H)**. **(A–D)** Microtiter wells showing the CV-stained biofilm phenotype at 24 h **(A)** and 48 h **(C)**, and their corresponding quantification at 24 h **(B)** and 48 h **(D)**. **(E–H)** A SYTO9- and PI-labelled fluorescent photomicrograph showing biofilm phenotypes at 24 h **(E)** and 48 h **(F)** and their corresponding thickness, as measured through 3D images from CLSM using a 10× objective lens at 24 h **(G)** and 48 h **(H)**. Mutant strains Δ*kdpD*::pUCP18 and Δ*aauS*::pUCP18 showed considerably reduced biofilm formation compared to the WT::pUCP18 strain, as determined using CV staining and fluorescence microscopic analysis. Similar to WT::pUCP18, the complemented strains (Δ*kdpD*::pUCP18*kdpD* and Δ*aauS*::pUCP18*aauS*) restored the biofilm, confirming the role of *kdpD* and *aauS* in the biofilm phenotype of *P. aeruginosa.* All the experiments were performed in triplicates. The significance of the data was analyzed using Student’s *t*-test. *P* < 0.05 was considered statistically significant (ns, non significant; *p < 0.05, **p < 0.01, and ***p < 0.005). The scale bar is 100 μm in confocal images.

KdpD/KdpE is an ‘adaptive regulator’ TCS involved in the virulence and intracellular survival of various pathogenic bacteria (Freeman et al., 2013). To elucidate the roles of *kdpD* and *aauS* in biofilm formation, we used a CV staining assay, followed by CLSM confirmation and image analysis to determine the quantity and thickness of the biofilm, respectively. Firstly, strains Δ*kdpD* and Δ*aauS* were tested against the WT (positive control) and Δ*pqsA (*negative control). After 24 and 48 h, Δ*pqsA* showed considerably poor biofilm formation compared to the WT. At 24 h, Δ*kdpD* and Δ*aauS* showed 69.1% and 54.9% reduction in biofilm formation, respectively (**Supplementary Figure S3A**). Similarly, we observed 69.7% and 72.8% inhibition of biofilm formation after 48 h in Δ*kdpD* and Δ*aauS*, respectively (**Supplementary Figure S3B**). Furthermore, knockouts were complemented with their corresponding genes in the pUCP18 vector to confirm the roles of *kdpD* and *aauS* in biofilm formation. Empty plasmid controls (pUCP18) were used for WT strains and knockouts. At 24 h, knockout strains with the empty plasmids Δ*kdpD*::pUCP18 and Δ*aauS*::pUCP18 showed 38.7% and 29.0% reduction in biofilm formation, respectively compared to WT::pUCP18 (**Figures 2A, B**). Similarly, Δ*kdpD*::pUCP18 and Δ*aauS*::pUCP18 showed 62.7% and 58.7% biofilm reduction, respectively at 48 h. The complemented strains Δ*kdpD*::pUCP18*kdpD*, and Δ*aauS*::pUCP18*aauS* showed biofilm formation similar to that of WT::pUCP18 (**Figures 2C, D**). These results indicate that *kdpD* and *aauS* contribute to biofilm formation. Furthermore, we investigated biofilm formation in the WT, Δ*kdpD*, Δ*aauS*, and complementation strains through CLSM using empty plasmid controls. At 24 and 48 h, Δ*kdpD*::pUCP18 and Δ*aauS*::pUCP18 showed reduced biofilm formation compared to WT::pUCP18, whereas their complemented strains Δ*kdpD*::pUCP18*kdpD* and Δ*aauS*::pUCP18*aauS* exhibited similar biofilm formation as WT::pUCP18 (**Figures 2E–H**). Based on these results, we concluded that *kdpD* and *aauS* play vital roles in the biofilm formation of *P. aeruginosa*.

### 3.3 Roles of *kdpD* and *aauS* in motility

**Figure 3:**
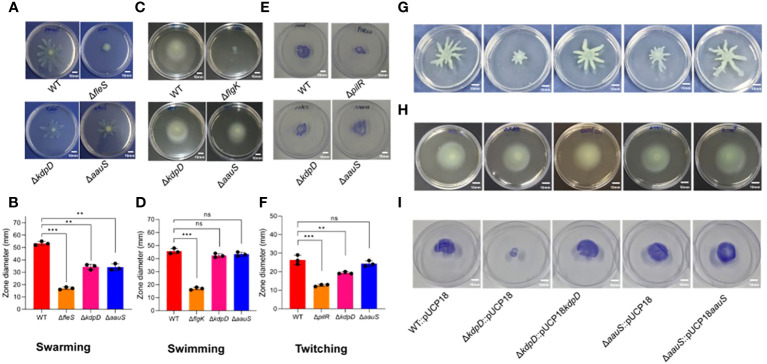
Assessment of the roles of *kdpD* and *aauS* in motility. Comparative qualitative and quantitative analysis of the motility phenotypes of Δ*kdpD* and Δ*aauS* mutant strains with the WT, including **(A, B)** swarming **(A)** and its quantification **(B)**; **(C, D)** swimming **(C)** and its quantification **(D)**; and **(E, F)** twitching **(E)** and its quantification **(F)**. The Δ*kdpD* and Δ*aauS* mutants with empty vectors (Δ*kdpD*::pUCP18, Δ*aauS*::pUCP18) were both complemented (Δ*kdpD*::pUCP18*kdpD* and Δ*aauS*::pUCP18*aauS*). The complemented strains showed a restoration of swarming **(G)**, swimming **(H)**, and **(I)** twitching motility similar to that of the WT *P. aeruginosa* containing an empty vector (WT::pUCP18*).* All the mobility assay experiments were conducted three times. The significance of the data was analyzed using Student’s *t*-test. *P* < 0.05 was considered statistically significant (ns, non significant; **p < 0.01, and ***p < 0.005). The scale bar is 10 mm in motility plates.

Bacterial motility is an important virulence factor in host-pathogen interactions. Therefore, we investigated the relevance of *kdpD* and *aauS* in motility by analyzing the comparative swarming phenotypes of the WT with Δ*kdpD*, Δ*aauS*, and mutant-complemented strains. Our data revealed markedly reduced swarming (69.7%) in Δ*fleS* compared to the WT (**Figures 3A, B**). Contrastingly, Δ*kdpD* and Δ*aauS* showed 37.5% and 35.8% reduction in swarming motility, respectively compared to the WT (**Figures 3A, B**). These results indicate that *kdpD* and *aauS* contribute to swarming motility.

The swimming phenotype is also important for flagellar motility, which plays an important role in infection by various pathogenic bacteria (Horstmann et al., 2017; Corral et al., 2020). Therefore, the involvement of *kdpD* and *aauS* was investigated to assess their roles in swimming motility. Here, Δ*flgK* showed a reduction of 62.9% in swimming compared to WT, whereas Δ*kdpD* and Δ*aauS* did not (**Figures 3C, D**). These findings suggest that these two genes are not involved in swimming motility in *P. aeruginosa*. Furthermore, we compared the twitching phenotypes of Δ*kdpD* and Δ*aauS* knockouts with those of the WT strain. We observed 62.9% and 26.5% reductions in twitching activity in Δ*pilR* and Δ*kdpD* compared to the WT. However, no reduction was observed in the twitching phenotype of the Δ*aauS* mutant strain (**Figures 3E, F**).

We confirmed these results by investigating the motility of WT::pUCP18, Δ*kdpD*::pUCP18, Δ*aauS*::pUCP18, Δ*kdpD*::pUCP18*kdpD*, and Δ*aauS*::pUCP18*aauS.* In this study, Δ*kdpD*::pUCP18 and Δ*aauS*::pUCP18 showed reduced swarming compared to the empty vector control (WT::pUCP18). In contrast, their corresponding mutant-complemented strains Δ*kdpD*::pUCP18*kdpD* and Δ*aauS*::pUCP18*aauS* exhibited a swarming phenotype similar to that of WT::pUCP18 (**Figure 3G**). These results indicate that *kdpD* and *aauS* contributory roles into the swarming mobility of *P. aeruginosa*. Complementation of the sensor kinases *aauS* and *kdpD* further confirmed that these genes have no role in swimming motility (**Figure 3H**). *aauS* displayed no role in twitching, whereas *kdpD* played a considerable role in twitching mobility. Similarly, Δ*kdpD*::pUCP18 showed reduced twitching, and Δ*kdpD*::pUCP18*kdpD* restored the phenotype to WT::pUCP18 (**Figure 3I**). These results indicate that *kdpD* contributes to swarming and twitching motility, whereas *aauS* only affects the swarming motility of *P. aeruginosa.*

### 3.4 The roles of *kdpD* and *aauS* in the inhibition of genes involved in biofilm and motility

**Figure 4:**
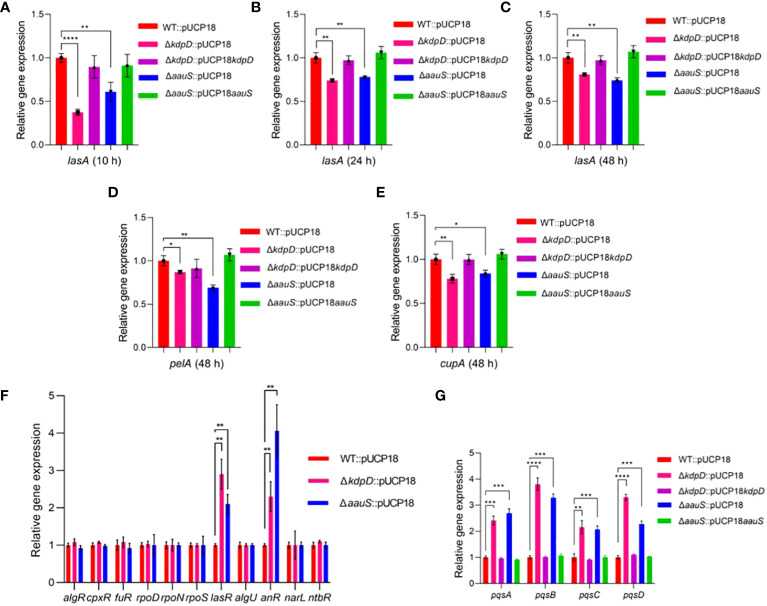
Quantitative-RT PCR analysis of the transcription factors (TFs) and their downstream genes in *kdpD* and *aauS* knockouts affecting biofilm formation, motility, and pathogenic potentials. **(A–C)** Relative expression of biofilm-regulated *lasA* in *kdpD*::pUCP18 and Δ*aauS*::pUCP18 mutants compared to WT::pUCP18 and their complemented Δ*kdpD*::pUCP18*kdpD* and Δ*aauS*::pUCP18*aauS* strains at 10, 24 and 48 h; **(D)** Relative expression of *pelA* in *kdpD*::pUCP18 and Δ*aauS*::pUCP18 mutants compared to WT::pUCP18 and their complemented Δ*kdpD*::pUCP18*kdpD* and Δ*aauS*::pUCP18*aauS* strains at 48 h; **(E)** Relative expression of *cupA* in *kdpD*::pUCP18 and Δ*aauS*::pUCP18 mutants compared to WT::pUCP18 and their complemented Δ*kdpD*::pUCP18*kdpD* and Δ*aauS*::pUCP18*aauS* strains at 48 h; **(F)** Relative gene expression of various transcription regulators (*algR*, *cpxR*, *fuR, rpoD, rpoN, rpoS, lasR, algU, anR, narL*, and *ntbR* in the *kdpD*::pUCP18 and Δ*aauS*::pUCP18 mutants) compared to the WT::pUCP18 strain; and **(G)** Relative gene expression of the PQS operon (*pqsA, pqsB pqsC* and *pqsD)* in Δ*kdpD*::pUCP18 and Δ*aauS*::pUCP18 mutants compared to the WT::pUCP18 strain and their complimented strains Δ*kdpD*::pUCP18*kdpD* and Δ*aauS*::pUCP18*aauS.* All the experiments were performed in triplicates. The significance of the data was analyzed using Student’s *t*-test. *P* < 0.05 was considered statistically significant (*p < 0.05, **p < 0.01, and ***p < 0.005, ****p < 0.0001).

Well-known genes were initially considered to assess the relative gene expression in Δ*kdpD* and Δ*aauS* knockouts with the empty plasmids to elucidate the role of *kdpD* and *aauS* in biofilm formation, swarming, and twitching mobility. Many genes play important roles in *P. aeruginosa* biofilm formation. For example, *lasA* plays a key role in biofilm development (Thi et al., 2020) while the *pelA* markedly contributes to the pellicle matrix formation of biofilm in *P. aeruginosa* (Friedman and Kolter, 2004; Sakuragi and Kolter, 2007; Colvin et al., 2012; Wei and Ma, 2013). Additionally, *cupA* is a crucial fimbria gene that contributes to biofilm formation *via* adhesion (Vallet et al., 2001; Vallet-Gely et al., 2007). Therefore, *lasA, pelA*, and *cupA* expression was investigated in the WT with empty plasmids and knockout with the empty plasmid strains. We found reduction in *lasA* expression at 10 h by 62.5% and 39.1% in Δ*kdpD*::pUCP18 and Δ*aauS*::pUCP18, respectively (**Figure 4A**). Complemented strains Δ*kdpD*::pUCP18*kdpD* and Δ*aauS*::pUCP18*aauS* restored expression levels to the level of of WT::pUCP18 (**Figure 4A**). Furthermore, *lasA* expression was also found to be reduced at 24 and 48 h time points by 25.9% and 19.1% in Δ*kdpD*::pUCP18; and 22.0% and 26.0% in case of Δ*aauS*::pUCP18, respectively (**Figures 4B, C**). These results demonstrate that *kdpD* and *aauS* contribute to initial biofilm formation by controlling *lasA* expression. We found no significant reduction in *pelA* and *cupA* in both the Δ*kdpD*::pUCP18 and Δ*aauS*::pUCP18 strains at 10 and 24 h (**Supplementary Figures S4A–D**). However, at 48 h *pelA* showed slightly reduced expression of 13.0% and 31.0% in the Δ*kdpD*::pUCP18 and Δ*aauS*::pUCP18 strains, respectively (**Figure 4D**). Furthermore, *cupA* also showed reduced expression of 21.9% and 15.3% at 48 h in the Δ*kdpD*::pUCP18 and Δ*aauS*::pUCP18 strains, respectively (**Figure 4E**). Complemented strains Δ*kdpD*::pUCP18*kdpD* and Δ*aauS*::pUCP18*aauS* restored expression levels to the level of WT::pUCP18 (**Figure 4E**). Overall, these results indicate that *kdpD* and *aauS* contribute to biofilm formation by modulating the expression of *lasA*, *pelA* and *cupA* genes.

Many genes and transcription regulators involved in flagellar biosynthesis, assembly and regulation are relevant to motility (Dasgupta et al., 2003). For example, *fleQ* is an important transcription regulator in *P. aeruginosa*, contributing to transcription of many flagellar genes (Arora et al., 1997). Similarly, FleS/FleR is a crucial TCS that modulates flagellar motility and adhesion (Ritchings et al., 1995). FleN is an anti-activator of FleQ, which plays a vital role in retaining a single flagellum (Dasgupta et al., 2000). In addition, sigma factor σ54 encoded by *rpoN* contributes to the regulation of flagellar expression (Totten et al., 1990). Furthermore, *flgM* is an important flagellar gene whose interactions with the sigma factor *fliA* contribute to flagellar biogenesis (Frisk et al., 2002). We analyzed the expression of several flagellar genes and transcription regulators in Δ*kdpD* and Δ*aauS* strains to investigate *kdpD* and *aauS* contribution to motility. There were no significant differences in the expression levels of the major motility-regulated genes *fleS*, *fleR*, *fleQ*, *flgK*, *fliC*, *fliD*, *flgD*, and *flgE* (**Supplementary Figure S5**). Therefore, a top-down approach was applied to comprehensively elucidate the mechanistic roles of *kdpD* and *aauS* in biofilm, motility, and pathogenesis. The expression of known transcription factors (TF) in Δ*kdpD*::pUCP18 and Δ*aauS*::pUCP18 compared to WT::pUCP18 was examined using q-RT-PCR. Accordingly, the transcriptional regulators (*algR*, *cpxR*, *furR*, *rpoD*, *rpoN*, *rpoS*, *lasR*, *algU*, *anR*, *narL*, and *nbtR*) that are directly or indirectly involved in biofilm formation, motility, and virulence gene expression have been assessed (Pusic et al., 2021). In this study, the expression levels of *lasR* was enhanced by 190% and 110% in Δ*kdpD*::pUCP18 and Δ*aauS*::pUCP18, respectively, compared to WT::pUCP18 (**Figure 4F**). Secondly, the *anR* expression was increased by 130% and 306% in Δ*kdpD*::pUCP18 and Δ*aauS*::pUCP18, respectively; compared to WT::pUCP18 (**Figure 4F**). *anR* and *lasR* contribute to biofilm formation and motility by controlling the *Pseudomonas* quinolone signal (PQS) quorum sensing (QS) system (Pusic et al., 2021). The *las*-QS system is essential for PQS-stimulated biofilm formation in *P. aeruginosa* PA14 (Christiaen et al., 2014). These results partially demonstrate that *kdpD* and *aauS* contribute to biofilm formation by controlling *lasA* expression through the *lasR* TF. PQS suppresses swarming motility in *P. aeruginosa* PA14 (Guo et al., 2014; García-Reyes et al., 2021). Therefore, we confirmed the expression levels of *pqs* in Δ*kdpD*::pUCP18 and Δ*aauS*::pUCP18. Our results showed increased *pqsA*, *pqsB*, *pqsC*, and *pqsD* expression (141%, 208%, 115% and 230%) in Δ*kdpD*::pUCP18, and (169%, 228%, 107% and 127%) in Δ*aauS*::pUCP18 strains (**Figure 4G**). Their expression was restored to that observed in WT::pUCP18 in the complemented (Δ*kdpD*::pUCP18*kdpD* and Δ*aauS*::pUCP18*aauS*) strains. Overall, these results indicate that *kdpD* and *aauS* contribute to swarming motility by altering the expression of the PQS quorum sensing system controlled by *anR* and *lasR* transcriptional regulators.

### 3.5 Δ*kdpD* and Δ*aauS* attenuate HeLa cell invasion and increased host cell survival

**Figure 5:**
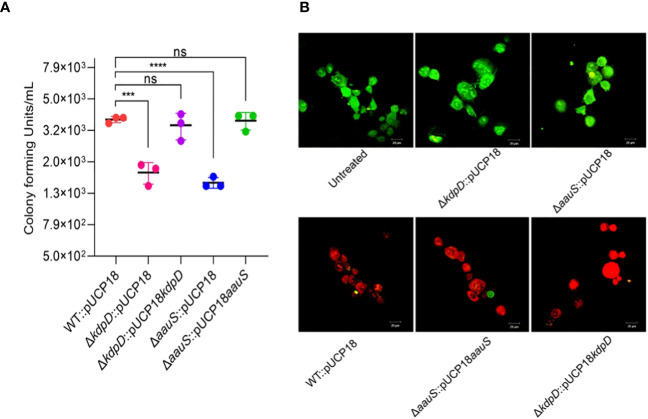
Role of *kdpD* and *aauS* in host cell survival in mammalian cell culture conditions. **(A)** Assessment of role of *kdpD* and *aauS* the internalization potential of using GPA. HeLa cells were infected with bacteria at an MOI of 10 for 120 min in a 5% CO_2_ environment at 37 **°**C. Extracellular bacteria were killed through gentamycin treatment, and internalized bacterial cells were assessed through CFU determination. **(B)** Assessment of the *in vitro* pathogenic potential of various strains using live/dead staining of the host-pathogen complex after infection, wherein HeLa cells were infected with WT::pUCP18, Δ*kdpD*::pUCP18, Δ*aauS*::pUCP18, Δ*kdpD*::pUCP18*kdpD*, and Δ*aauS*::pUCP18*aauS* at an *MOI* of 10. Untreated cells were used as negative controls. After 2 h infection, the cells were stained with PI (red fluorescence) and fluorescein diacetate (green fluorescence) and visualized using CLSM. Green represents live cells, whereas red represents dead cells. Knockout strains of *kdpD* and *aauS* with empty vectors (Δ*kdpD*::pUCP18, Δ*aauS*::pUCP18) were the least toxic to host cells compared to the wild-type with empty vectors (WT::pUCP18) and complemented strains (Δ*kdpD*::pUCP18*kdpD* and Δ*aauS*::pUCP18*aauS*). The experiments were performed in triplicates. The significance of the data was analyzed using Student’s *t*-test. *P* < 0.05 was considered statistically significant (ns, non significant; ***p < 0.005, ****p < 0.0001). The scale bar is 20 μm in live dead images.

*P. aeruginosa* mainly invades mammalian epithelial cells via the type III secretion system (T3S) (Hernández-Padilla et al., 2017; Kroken et al., 2018). Therefore, we investigated the invasive activity of mutant strains with empty plasmids to evaluate the roles of *kdpD* and *aauS* in cell invasion. HeLa cells were infected with bacteria at an MOI of 10 for 120 min, and the number of intracellular bacterial cells was measured through CFU enumeration. Strains Δ*kdpD*::pUCP18 and Δ*aauS*::pUCP18 showed reduced invasion activity, with intracellular CFUs difference of 2.0*×*10^3^ and 2.3×10^3^ CFU/mL, respectively compared to WT::pUCP18. In contrast, the intracellular CFU of the WT::pUCP18 strain was 3.7×10^3^ CFU/mL (**Figure 5A**). Moreover, the complemented strains Δ*kdpD*::pUCP18*kdpD* and Δ*aauS*::pUCP18*aauS* restored the intracellular CFU count to that of WT::pUCP18.

The host-pathogen complex was stained with live/dead (green/red) reagents to further evaluate the roles of *kdpD* and *aauS* in HeLa cell infection. Uninfected HeLa cells mostly retained green fluorophores (Untreated, **Figure 5B**). A high number of dead host cells was observed when WT::pUCP18 was added (**Figure 5B**). Moreover, a markedly increased number of live cells was observed when host cells were infected with Δ*kdpD*::pUCP18 or Δ*aauS*::pUCP18 (**Figure 5B**). Contrastingly, a considerable number of dead host cells was observed when the complemented strains Δ*kdpD*::pUCP18*kdpD* and Δ*aauS*::pUCP18*aauS* were used to infect HeLa cells. These results demonstrate the involvement of *kdpD* and *aauS* in host cell invasion and infection.

### 3.6 *kdpD* and *aauS* play critical roles in *G. mellonella* infection

**Figure 6:**
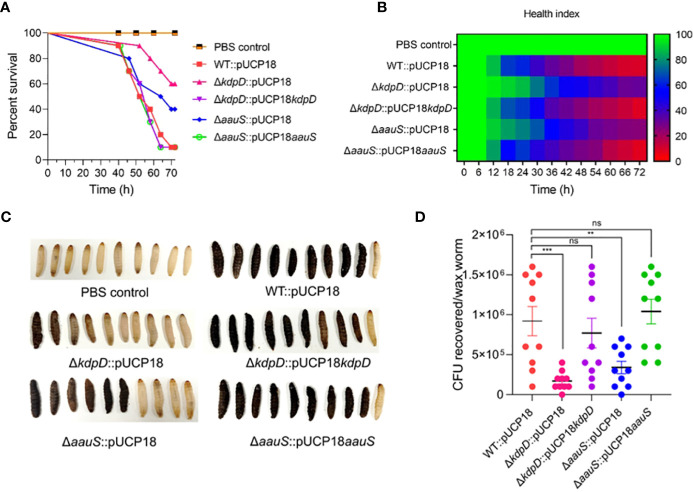
Confirming the roles of *kdpD* and *aauS* in the virulence potential of *P. aeruginosa* using the *G. mellonella* infection model. **(A)** Survival of *G. mellonella* larvae infected with 10 CFU of WT::pUCP18, Δ*kdpD*::pUCP18, Δ*aauS*::pUCP18, Δ*kdpD*::pUCP18*kdpD*, and Δ*aauS*::pUCP18*aauS* strains. The number of larvae in each group was 10 (n =10). The larvae were injected with 20 µL bacterial solution, and their survival was examined for 72 (h) The control group received 20 µL PBS. **(B)** The health index of larvae is plotted as an average of each group at multiple time points based on the health scoring index (movement, melaninization, cocoon formation, and survival). **(C)** An image showing the differential melaninization and death of waxworms infected with different bacterial strains; and **(D)** Assessment of CFUs recovered on selective media CA after 72 h of infection. The experiments were performed in triplicates. The significance of the data was analyzed using Student’s *t*-test. *P* < 0.05 was considered statistically significant (ns, non significant; **p < 0.01, and ***p < 0.005).

*G. mellonella* larvae were challenged with WT::pUCP18, Δ*kdpD*::pUCP18, Δ*aauS*::pUCP18, Δ*kdpD*::pUCP18*kdpD*, and Δ*aauS*::pUCP18*aauS* at 10 CFU/larvae to further analyze the roles of *kdpD* and *aauS* in virulence. The WT::pUCP18 bacterial load was designed to allow 10% survival 72 h post-infection. Then, survival percentages of the larvae and their health indices were recorded. Our results indicated delayed mortality, with increased survival in Δ*kdpD*::pUCP18 (60%) and Δ*aauS*::pUCP18 (40%) compared to WT::pUCP18, after 72 h. This is consistent with the results obtained in the initial screening (**Figure 1**). We further investigated the infection properties of complemented Δ*kdpD*::pUCP18*kdpD* and Δ*aauS*::pUCP18*aauS* strains. The results showed a survival comparable to that of WT::pUCP18 cells (**Figure 6A**). We also recorded the health index of each strain based on movement, cocoon formation, melaninization, and survival. Our results showed greater movement, full cocoon formation with less melaninization, and increased survival in larvae infected with Δ*kdpD*::pUCP18 and Δ*aauS*::pUCP18 than in those infected with WT::pUCP18. However, larvae infected with Δ*kdpD*::pUCP18*kdpD* and Δ*aauS*::pUCP18*aauS*showed less movement, greater melaninization, less cocoon formation, and decreased survival 72 h post-infection, demonstrating results similar to those of WT::pUCP18 (**Figures 6B, C**). Next, we determined the bacterial burden in the worms by measuring the number of bacteria in the infected larvae. Larvae infected with Δ*kdpD*::pUCP18 and Δ*aauS*::pUCP18 showed 1.7*×*10^5^ and 3.4*×*10^5^CFU/mL, respectively. These were lower than the 9.3*×*10^5^ CFU/mL observed in the WT::pUCP18 strain (**Figure 6D**). However, strains Δ*kdpD*::pUCP18*kdpD* and Δ*aauS*::pUCP18*aauS* showed no significant reduction in CFU compared to WT::pUCP18 (**Figure 6D**). These results were consistent with the survival and health index results, indicating that *kdpD* and *aauS* play important roles in *G. mellonella* infection.

## 4 Discussion

*P. aeruginosa* is a gram-negative environmental MDR pathogen responsible for acute and chronic diseases, owing to its extensive biofilm formation. Protease, elastase, and phospholipase (Vasil, 1986) determine *P. aeruginosa* biofilm production, motility, pigment secretion, and pathogenic potential. These virulence determinants play important roles in bacterial colonization and host tissue invasion, which may lead to life-threatening infections. Chronic infections caused by biofilm formation include chronic pneumonia in patients with CF, colonization of ventilators and urinary catheters, and infected burn wounds (Bjarnsholt, 2013). Moreover, pseudomonad biofilm is a major cause of antimicrobial therapy failure because the biofilm enhances antimicrobial resistance by 2–3 log values (Yin et al., 2022).

*P. aeruginosa* uses TCSs to sense signals and coordinate bacterial virulence. The sensor HKs and RRs considerably contribute to *P. aeruginosa* metabolism and pathophysiology. However, only a few of these TCSs have been studied to investigate their role in virulence. Therefore, we screened a few *P. aeruginosa* TCSs whose functions in virulence are not clearly understood. We assessed the virulence potential of their mutant strains and identified two HKs that are involved in *P. aeruginosa* virulence. Furthermore, we investigated and confirmed the roles of these TCSs in the biofilm formation and motility of *P. aeruginosa*.

KdpD senses environmental signals to activate the expression of the K^+^ transport system in *E. coli* (Epstein, 2016). Previous studies have emphasized its role in the virulence of other bacteria (Zhao et al., 2010; Xue et al., 2020). KdpD/KdpE is responsible for the virulence and motility of avian pathogenic *E. coli* (Xue et al., 2020). This TCS regulates the transcription of the flagellar-associated genes *fliR* and *fliP*. The deletion of KdpD/KdpE modulates the expression of several genes, including *cap* (synthesis of capsule), *hla* (alpha-toxin), *spa* (surface protein), *geh* (lipase gene), and *hlgB* (gamma-hemolysin), in *S. aureus* (Zhao et al., 2010). The AauS/AauR TCS is involved in acidic amino acid utilization in *Pseudomonas putida* (Sonawane and Singh, 2006). In *P. putida*, Δ*aauS* and Δ*aauR* cannot consume glutamate, aspartate, or glutamine as the sole nitrogen and carbon sources (Sonawane and Singh, 2006). Additionally, KdpD/KdpE regulation responds to virulence-related conditions, including phagocytosis, exposure to microbicides, host hormones, and quorum sensing (QS) signals (Freeman et al., 2013; Ali et al., 2017). However, the role of *aauS* in the virulence of other bacteria, including *P*. *aeruginosa* has not been intensively investigated until now. Therefore, our results provide clear evidence of *kdpD* and *aauS*-mediated *P. aeruginosa* virulence.

Several TCSs contribute to biofilm formation in *P. aeruginosa* by sensing signals, eventually causing the bacteria to enter the sessile phase (Mikkelsen et al., 2011). GacS/GacA acts along with RetS and LadS to regulate the switch between planktonic and biofilm lifestyles (Mikkelsen et al., 2011; Chambonnier et al., 2016). Biofilm formation occurs at different stages, with BfiS/BfiR, MifS/MifR, and BfmS/BfmR playing essential roles in irreversible attachment, microcolony formation, and maturation of biofilm (Petrova and Sauer, 2009). Furthermore, microarray analysis and promoter fusion confirmed the role of FleSR in biofilm formation (Dasgupta et al., 2003). Our quantitative biofilm results and confocal microscopy data showed *kdpD* and *aauS* involvement in the regulation of biofilm formation (**Figure 2**). Therefore, in addition to known biofilm-associated TCSs, we propose that KdpD/KdpE and AauR/AauS are responsible for biofilm formation. In *P. aeruginosa*, QS systems play vital roles in biofilm formation (Solano et al., 2014; Thi et al., 2020). Therefore, the HK KdpD sensor could activate its cognate RR to express the genes involved in biofilm formation. Our gene expression results showed reduced expression of *pelA*, *cupA*, increased *lasR* TF expression and reduced expression of the downstream gene *lasA* in Δ*kdpD*::pUCP18 and Δ*aauS*::pUCP18, confirming that *kdpD* and *aauS* involvement in biofilm formation occurs via the *pelA*, *cupA* and *las*-QS system (**Figures 4A–E**). The role of KdpD and AauS in *P. aeruginosa* biofilm is consistent with study of Badal et al. (2020) wherein KdpD and AauS was identified to involve in biofilm formation of *P. aeruginosa* on endotracheal tubes (Badal et al., 2020).

The pathogenesis of *P. aeruginosa* depends on motility, which modulates its mobilization and colonization during biofilm formation (O'Toole and Kolter, 1998), as demonstrated in studies investigating *P. fluorescens* and *E. coli* (Korber et al., 1994; Pratt and Kolter, 1998). Many TCSs and genes are associated with swarming, swimming, and twitching motility (Sultan et al., 2021). For example, FleS/FleR regulates the expression of flagellar biosynthetic genes and alters swarming motility. Similarly, CarS/CarR senses and modulates Ca^2+^ homeostasis. This crucial TCS further contributes to swarming motility via *carP* (Guragain et al., 2016). PilS/PilR is another important TCS responsible for twitching and flagellum-dependent swimming motility (Kilmury and Burrows, 2018a). As we did not observe any changes in *pilS*, *pilR*, *carS*, or *carR* in the *kdpD* or *aauS* knockout strains, their contribution to motility seemed to be independent of PilS/PilR or CarS/CarR involvement. Similarly, we did not observe any mechanistic role of *kdpD* and *aauS* in motility by FleS/FleR, as there were no significant differences in the expression of flagellar genes. The metabolic regulator *anR* and quorum-sensing regulator *lasR* control the PQS system to regulate biofilm formation and motility (Pusic et al., 2021). PQS suppresses swarming motility in *P. aeruginosa* PA14 (Guo et al., 2014; García-Reyes et al., 2021). In this study, *anR* and *lasR* expression was increased in *kdpD*::pUCP18 and Δ*aauS*::pUCP18 knockouts, confirming the roles of *kdpD* and *aauS* in swarming motility, which is conferred by these transcription regulators. The expression of PQS genes increases when *anR* and *lasR* expression is upregulated (Pusic et al., 2021). In our study, *pqsA, pqsB, pqsC*, and *pqsD* expression was also increased in Δ*kdpD*::pUCP18 and Δ*aauS*::pUCP18 knockouts (**Figure 4G**). This expression was restored to the level of WT::pUCP18 in the complement strains Δ*kdpD*::pUCP18*kdpD* and Δ*aauS*::pUCP18*aauS*. Overall, these results confirmed that *kdpD* plays a role in swarming motility by altering the expression of PQS quorum sensing system controlled by *anR* and *lasR* transcription regulators.

Several swarming-deficient mutants of *P. aeruginosa* showed poor biofilm formation, suggesting a strong relationship between swarming and biofilm formation (Overhage et al., 2007). Consistent with this finding, *kdpD* and *aauS* were associated with the swarming phenotype and biofilm formation in our study. However, twitching activity was not altered by *aauS* but was affected by *kdpD.* Furthermore, *kdpD* and *aauS* exhibited no roles in swimming motility. These results suggest that KdpD and AauS sensor HKs contribute to *P. aeruginosa* virulence through swarming motility.

In this study, we identified the roles of *kdpD* and *aauS* in the biofilm formation and motility of *P. aeruginosa via* several key genes involved in these virulence phenotypes. These roles were validated through cell invasion (**Figure 5**) and a *G. mellonella* infection model (**Figure 6**). The effects of *kdpD* and *aauS* were minimal on phenotype reduction in their knockout strains compared with the genes used as controls, such as *pqsA* for biofilm formation and *fleS, flgK*, and *pilR* for motility. However, our results clearly demonstrate the contributions of *kdpD* and *aauS* to the virulence phenotype. This was confirmed by the reduced infection activity of the knockout strain in the infection model and reduced expression of genes that control virulence. Therefore, we propose that *kdpD* and *aauS* are virulence-controlling TCSs. This study represents a milestone in the development of methods for identifying and validating key genes involved in bacterial virulence. Furthermore, our findings provide a molecular basis for understanding the virulence of *P. aeruginosa* and contribute to the design of inhibitors that target TCSs to overcome biofilm-mediated AMR.

## Data Availability

The original contributions presented in the study are included in the article/**Supplementary material**. Further inquiries can be directed to the corresponding authors.
