# Corrigendum: Sensor histidine kinases *kdpD* and *aauS* regulate biofilm and virulence in *Pseudomonas aeruginosa* PA14

**DOI:** 10.3389/fcimb.2024.1501233

**Published:** 2024-10-16

**Authors:** Maria Sultan, Rekha Arya, Akhilesh Kumar Chaurasia, Kyeong Kyu Kim

**Affiliations:** ^1^ Department of Precision Medicine, Graduate School of Basic Medical Science, Institute for Antimicrobial Resistance Research and Therapeutics, Sungkyunkwan University School of Medicine, Suwon, Republic of Korea; ^2^ Department of Orthopedic Surgery, University of Pittsburgh School of Medicine, Pittsburgh, PA, United States

**Keywords:** *Pseudomonas aeruginosa*, two-component system (TCS), sensor histidine kinase, KdpD, AauS, quorum sensing (QS), virulence

In the originally published article, a part of **Figure 6** has duplication of waxworm image **Figure 6C** and editing errors in the strain names **Figure 6D** while making the final high-resolution images.

**Figure 6:**
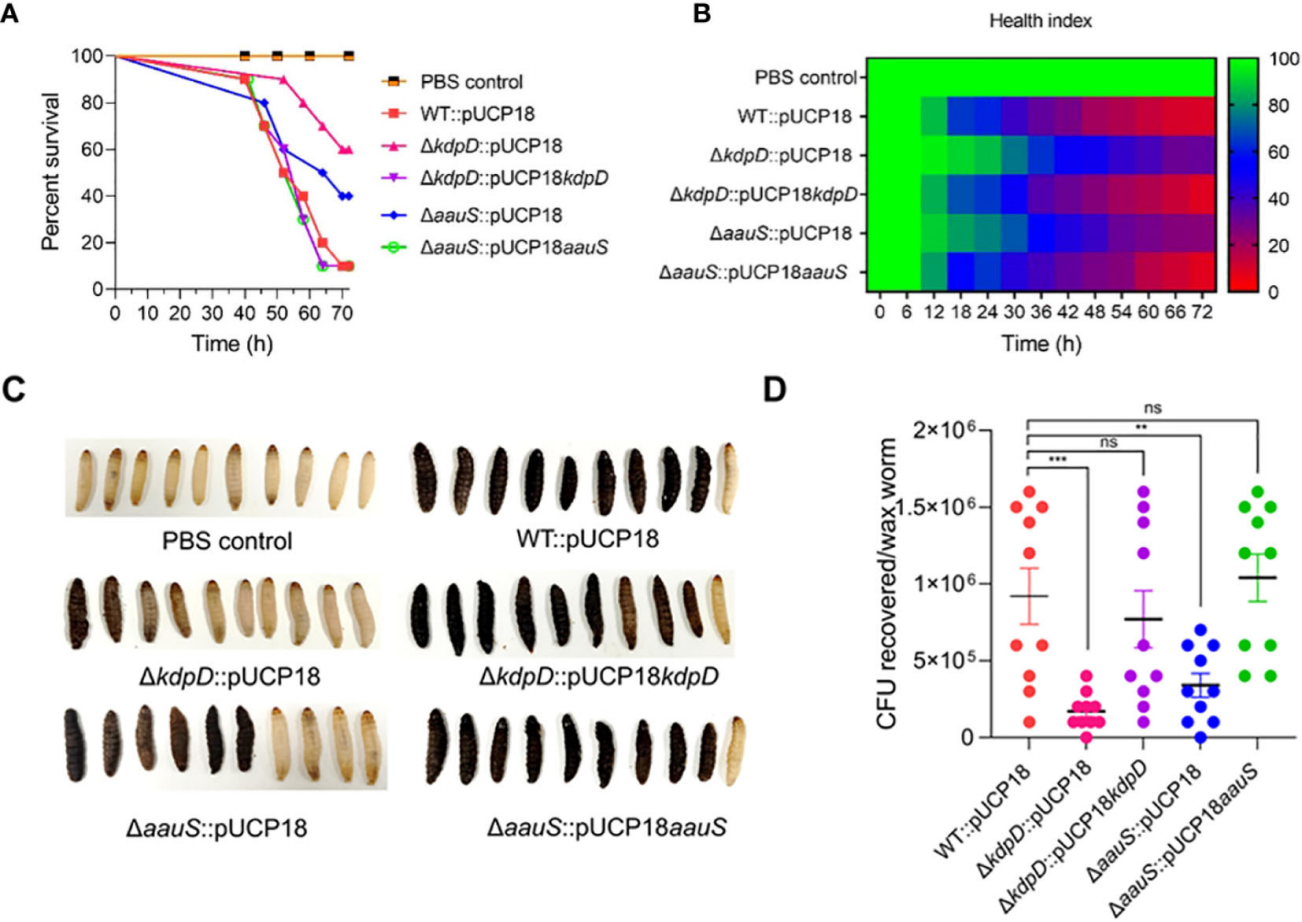
Confirming the roles of *kdpD* and *aauS* in the virulence potential of *P. aeruginosa* using the *G. mellonella* infection model. **(A)** Survival of *G. mellonella* larvae infected with 10 CFU of WT::pUCP18, Δ*kdpD*::pUCP18, Δ*aauS*::pUCP18, Δ*kdpD*::pUCP18*kdpD*, and Δ*aauS*::pUCP18*aauS* strains. The number of larvae in each group was 10 (n =10). The larvae were injected with 20 µL bacterial solution, and their survival was examined for 72 (h) The control group received 20 µL PBS. **(B)** The health index of larvae is plotted as an average of each group at multiple time points based on the health scoring index (movement, melaninization, cocoon formation, and survival). **(C)** An image showing the differential melaninization and death of waxworms infected with different bacterial strains; and **(D)** Assessment of CFUs recovered on selective media CA after 72 h of infection. The experiments were performed in triplicates. The significance of the data was analyzed using Student’s t-test. *P* < 0.05 was considered statistically significant (ns, non significant; **p < 0.01, and ***p < 0.005).

Specifically, the worm image in two panels of **Figure 6C** (PBS control-WT pUCP18 and Δ*kdpD*::pUCP18-Δ*kdpD*::pUCP18*kdpD*) are duplicated. The names of two strains (Δ*kdpD*::pUCP18*kdpD* and Δ*aauS*::pUCP18) in **Figure 6D** are interchanged. These errors have been corrected as shown in **Figures 6C, D** below. There is no change in the figure legends. We apologize for any inconvenience this error may have caused. It is noteworthy that these modifications do not change the scientific conclusions of the paper in any way.

