# Supplementary material for: Sensor histidine kinases kdpD and aauS regulate biofilm and virulence in Pseudomonas aeruginosa PA14
Source: Front Cell Infect Microbiol. 2023 Oct 10;13:1270667. doi: 10.3389/fcimb.2023.1270667 (PMC10595159; doi:10.3389/fcimb.2023.1270667)
Supplement: Supplementary file 1 [file DataSheet1.pdf]

SUPPORTING INFORMATION

Title

Sensor histidine kinases *kdpD* and *aauS* regulate biofilm and virulence in *Pseudomonas aeruginosa* PA14.

Running Title

kdpD and *aauS* regulate virulence in PA14.

Authors

Maria Sultan, Rekha Arya*, Akhilesh Kumar Chaurasia*, Kyeong Kyu Kim*

Affiliation

Department of Precision Medicine, Graduate School of Basic Medical Science, Institute for Antimicrobial Resistance Research and Therapeutics, Sungkyunkwan University School of Medicine, Suwon 16319, Republic of Korea.

***Corresponding authors:**

Kyeong Kyu Kim, Rekha Arya, and Akhilesh Kumar Chaurasia

Email: kyeongkyu@skku.edu

Tel.: +82-31-299-6152

Fax: +82-31-299-6159

TABLE OF CONTENTS

1. Preliminary Screening of Infection Potentials of Knockout of Uncharacterized Sensor Kinases and Response Regulators in *P. aeruginosa* using *Galleria mellonella* Infection Model.
2. Comparative Growth Profile of Knockouts with Wild Type PA14 Strains.
3. Biofilm Potential of Δ*kdpD* and Δ*aauS* Knockout Strains Compared to Wild Type and Δ*pqsA* Knockout Strains.
4. Quantitative-RT PCR Analysis of Genes in Δ*kdpD* and *ΔaauS* Knockouts Affecting Biofilm.
5. Quantitative-RT PCR Analysis of Major Motility Genes in Δ*kdpD* and *ΔaauS* Knockouts.
6. Supplementary Table S1: Strains and Plasmids used in this study.
7. Supplementary Table S2: Primers used in the study.
8. References

1. SUPPLEMENTARY FIGURE 1

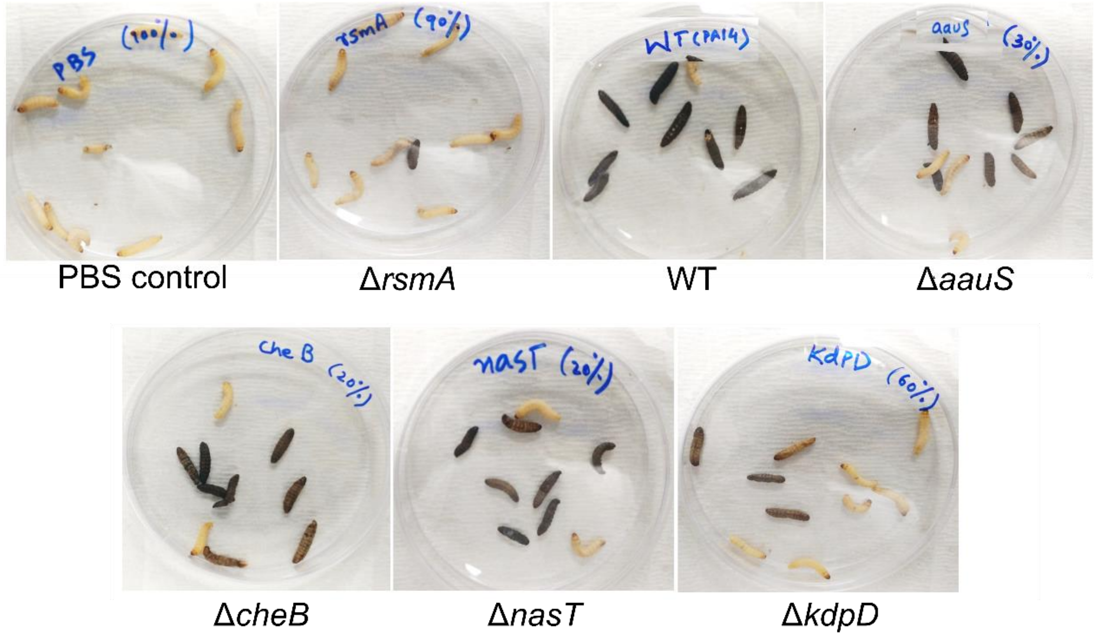

SUPPLEMENTARY FIGURE 1 | Preliminary screening of infection potentials of knockout of uncharacterized sensor kinases and response regulators in *P. aeruginosa* using *Galleria mellonella* infection model. The picture showing the infected waxworm with WT, Δ*rsmA,* Δ*kdpD*, Δ*aauS*, Δ*nasT,* or Δ*cheB*. Ten colony-farming units (CFUs) for each strain were injected into waxworms (*n* =10), followed by incubation at 27 °C to examine the survival for up to 72 h. A delay in mortality was observed for Δ*kdpD* and Δ*aauS,* with 60% and 30% survival, respectively.

2. SUPPLEMENTARY FIGURE 2

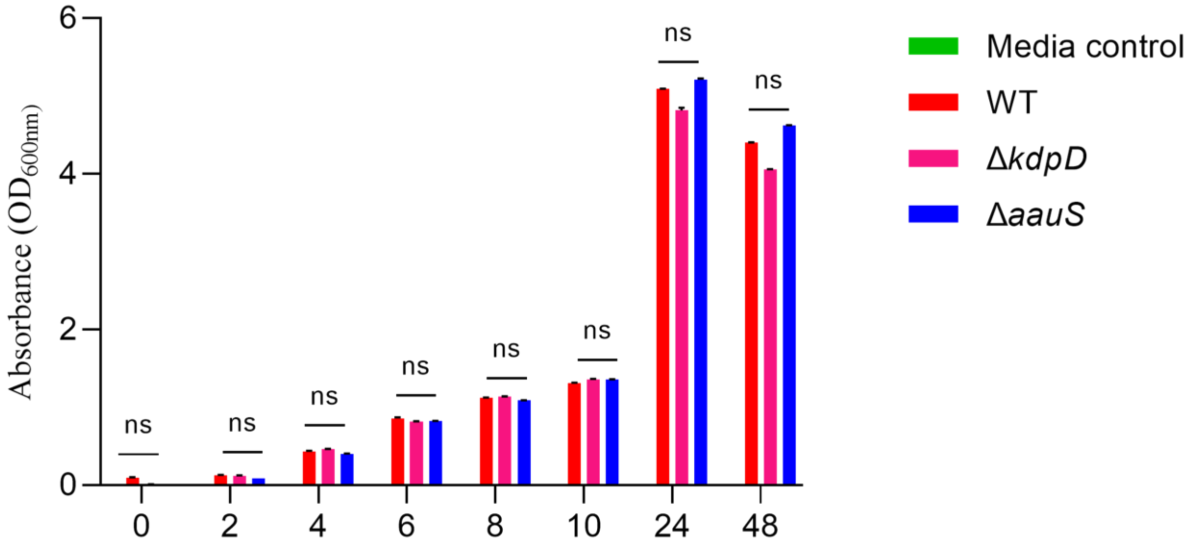

SUPPLEMENTARY FIGURE 2 | Comparative growth profiles of knockouts and WT strains. The overnight-grown cultures of WT, Δ*kdpD,* and Δ*aauS strains were* inoculated *(*1 µL) in 100 µL fresh LB medium and incubated at 37 °C for 48 h to assess their comparative growth. The growth of these strains was recorded spectrophotometrically by measuring the optical density (OD) at 600 nm at. The three strains exhibited identical growth profiles.

3. SUPPLEMENTARY FIGURE 3

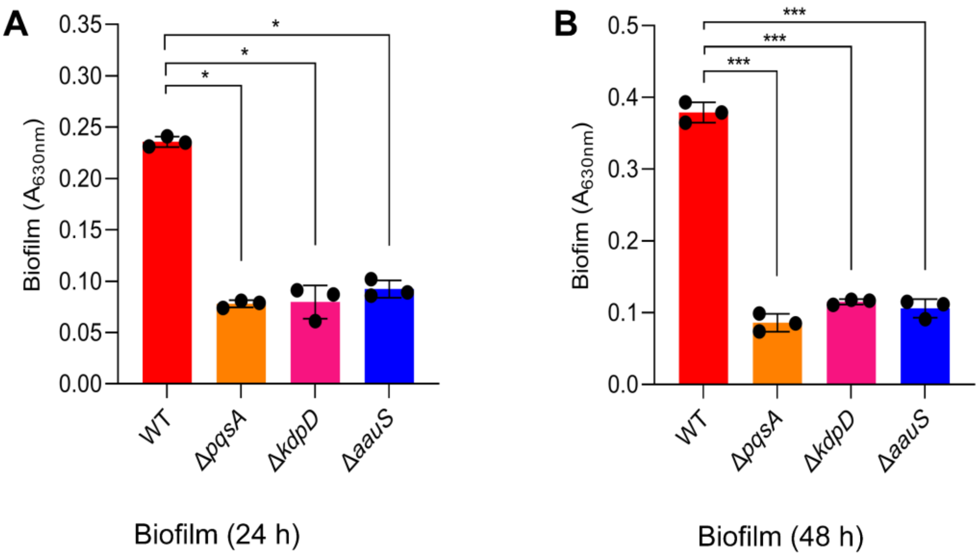

SUPPLEMENTARY FIGURE 3 | Biofilm potential of Δ*kdpD* and Δ*aauS* knockout strains compared to wild-type (WT) and Δ*pqsA* knockout strains was determined using 96-well polystyrene microtiter plates. Quantification of biofilms at **(A)** 24 h and (**B**) 48 h. The experiment was performed in triplicates. The significance of the data was analyzed using Student's *t*-test. $P < 0.05$ was considered statistically significant (* $p < 0.05$, ** $p < 0.01$, and *** $p < 0.005$). Both Δ*kdpD*-and Δ*aauS* knockout strains displayed reduced biofilm formation. The decrease in biofilm levels in Δ*kdpD* and Δ*aauS* knockouts was in the same range as that in the positive control possessing a well-known biofilm-controlling gene knockout strain, Δ*pqsA*. These results clearly indicate the involvement of *kdpD* and *aauS* in the biofilm phenotypes of *P. aeruginosa.*

4. SUPPLEMENTARY FIGURE 4

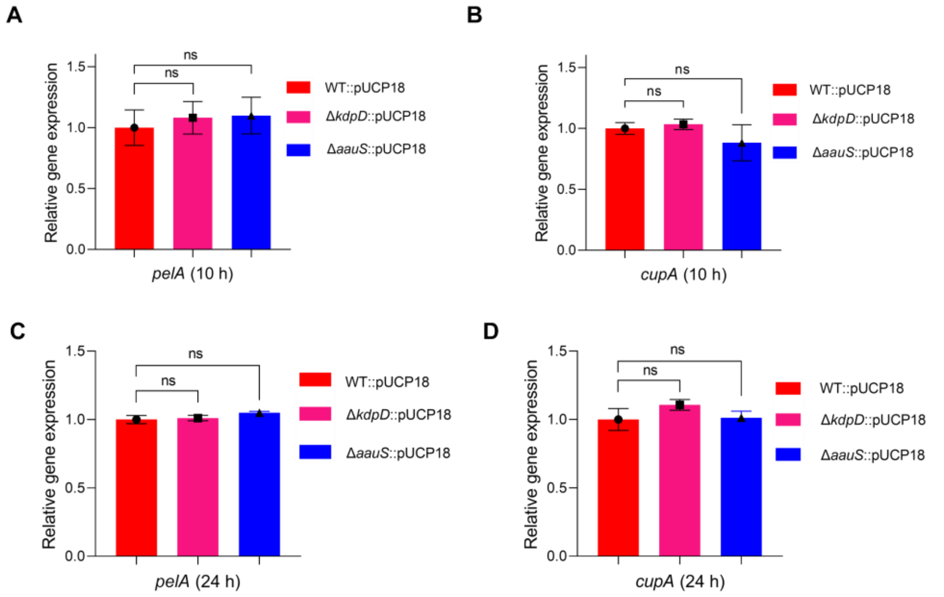

SUPPLEMENTARY FIGURE 4 | Quantitative RT-PCR analysis of genes in Δ*kdpD*::pUCP18 and Δ*aauS*::pUCP18 strains affecting biofilm. **(A)** Relative *pelA* expression in Δ*kdpD*::pUCP18 and Δ*aauS*::pUCP18 strains compared to the WT::pUCP18 at 10 h; **(B)** Relative *cupA* expression in Δ*kdpD*::pUCP18 and Δ*aauS*::pUCP18 strains compared to the WT::pUCP18 at 10 h. **(C)** Relative *pelA* expression in Δ*kdpD*::pUCP18 and Δ*aauS*::pUCP18 strains compared to the WT::pUCP18 at 24 h; **(D)** Relative *cupA* expression in Δ*kdpD*::pUCP18 and Δ*aauS*::pUCP18 strains compared to the WT::pUCP18 at 24 h. All the experiments were performed in triplicates. The significance of the data was analyzed using Student's *t*-test. $P < 0.05$ was considered statistically significant (* $p < 0.05$, ** $p < 0.01$, and *** $p < 0.005$). *pelA* and *cupA* are involved in biofilm formation in *P. aeruginosa*. There was no significant change in the expression of these genes in Δ*kdpD*::pUCP18 and Δ*aauS*::pUCP18 strains compared to the WT::pUCP18 strain. These results show that Δ*kdpD* and Δ*aauS* knockouts did not affect the expression of *pelA* and *cupA* mRNA at 10 and 24 h of biofilm formation. Therefore, the reduction in biofilm potential in the Δ*kdpD* and Δ*aauS* knockout strains appears to follow a novel regulatory cascade(s).

5. SUPPLEMENTARY FIGURE 5

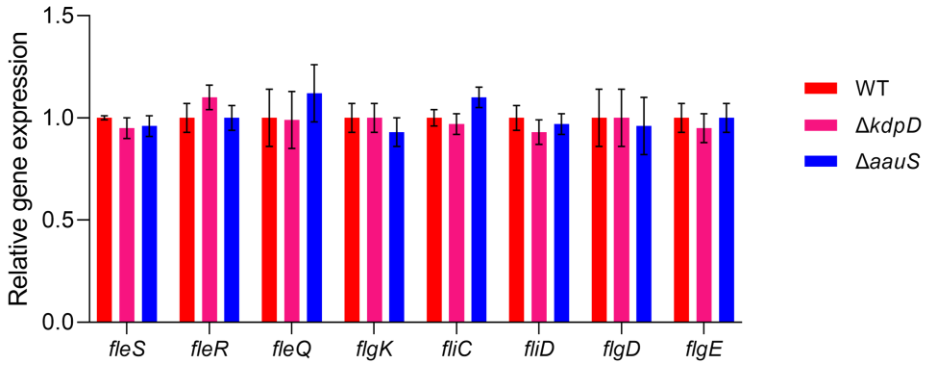

SUPPLEMENTARY FIGURE 5 | Quantitative RT-PCR analysis of major motility genes in Δ*kdpD* and *ΔaauS* knockouts. Relative expression of various major motility-regulated genes, namely *fleS*, *fleR*, *fleQ*, *flgK*, *fliC*, *fliD*, *flgD,* and *flgE* in Δ*kdpD* and Δ*aauS knockouts* compared to the WT. Expression of these genes was identical in both knockouts compared to the WT. These results indicate the involvement of new genes or regulatory cascades through which the motility of Δ*kdpD* and Δ*aauS* knockouts is affected.

6. SUPPLEMENTARY TABLE S1. Strains and plasmids used in the study.

Strains/Plasmids	Descriptions	Locus name	Reference
**Strains**			
Escherichia coli DH5α	Strain for cloning and plasmid amplification		Invitrogen, USA
PA14WT	*P. aeruginosa* wild type (WT)		(Liberati et al., 2006)
kdpD	Two-component sensor (K^+ transport)	PA14_43350	(Liberati et al., 2006)
aauS	Putative-two-component sensor (acidic amino acid utilization)	PA14_46980	(Liberati et al., 2006)
nasT	Response regulator (hypothetical protein)	PA14_41490	(Liberati et al., 2006)
cheB	Response regulator	PA14_02180	(Liberati et al., 2006)
rsmA	Regulator of secondary metabolites	PA14_52570	(Liberati et al., 2006)
pqsA	Probable coenzyme A ligase	PA14_51430	(Liberati et al., 2006)
fleS	Two-component sensor	PA14_50200	(Liberati et al., 2006)
flgK	Flagellar-hook-associated protein 1 FlgK	PA14_50360	(Liberati et al., 2006)
pilR	Type IV B pilus Protein	PA14_59310	(Liberati et al., 2006)
**Plasmids**			
Plasmid pUCP18	*P. aeruginosa-E. coli* shuttle vector		(Schweizer, 1991)
WT::pUCP18	WT with empty vector pUCP18		This study
Δ*kdpD*::pUCP18	Δ*kdpD* with empty vector pUCP18		This study
Δ*aauS*::pUCP18	Δ*aauS* with empty vector pUCP18		This study
Δ*kdpD*::pUCP18*kdpD*	Δ*kdpD* with recombinant vector pUCP18		This study
Δ*aauS*::pUCP18*aauS*	Δ*aauS* with recombinant vector pUCP18		This study

7. **SUPPLEMENTARY TABLE S2. List of primers used in the study.**

**Purpose**	**Name**	**Sequence** (5' to 3')
**Cloning**	*KdpD*-EcorI-fwd	ACTATAGAATTCATGACCGACACCGCAC
	kdpD-HindIII-rev	ACTATAAAGCTTTCATTCGTCGCCCTCC
	aauS-EcorI-fwd	ACTATAGAATTCATGGAATCCCCCGACCC
	aauS-HindIII-rev	ACTATAAAGCTTTCATCGGGCGGGACTC
**qRT-PCR**	*rho*-fwd	ACGAGGTGATCTACGAGGAAT
	rho-rev	GTACCCGAACGGTTGATGTT
	lasA-fwd	GAATGACGACCTGTTCCTCTAC
	lasA-rev	CTCCAGGTATTCGCTCTTGTC
	pqsA-fwd	CCCTTTGCTCGACGATTTCT
	pqsA-rev	GTGGAACCCGAGGTGTATTG
	pqsB-fwd	CTCTACGTGGTCGATACCCT
	pqsB-rev	TCGCTGTCCACTTCCAATC
	pqsC-fwd	TTGGATTCGCAGATGGAGTG
	pqsC-rev	ACACCACCAGCACTTTCTC
	pqsD-fwd	GTCAGCAATCATGACCTGGTAG
	pqsD-rev	TTCCGGCTCGACGTGATA
	algR-fwd	TTCATTGCCGACCACAAGTA
	algR-rev	GTCTTCCAGCGCCTTCAA
	cpxR-fwd	ATGAGCGAACTGCTGTTGA
	cpxR-rev	ACGCACGGAGAAACCTTC
	fuR-fwd	TGGATGCGGAAATCGAGAAG
	fuR-rev	CACGTAGAGCACCAGATTGT
	rpoD-fwd	TGATCCAGGAAGGCAACATC
	rpoD-rev	AGGTGGCGTAGGTAGAGAAT
	rpoN-fwd	ACAGCAAGATCGCTGGTTTA
	rpoN-rev	CCAGGGATTCGCGGTATTT
	rpoS-fwd	CGGAGTTTGACCACGATGAT
	rpoS-rev	GAGAAGGAAGTGGTGGCTTT
	lasR-fwd	CTGTGGATGCTCAAGGACTAC

lasR-rev	CCACTGCAACACTTCCTTCT
algU-fwd	CAGGAACAGGATCAGCAACT
algU-rev	CGCACGATCAATCCCAGTAT
anR-fwd	GCAACGAGATCGGCAACTA
anR-rev	TCGATGGAGTCGAGGATGT
narL-fwd	CTGCTGGACCTGAACATGAA
narL-rev	ACGGTGAACACCACGATG
ntbR-fwd	CTCCAGGAAACCATCTTCTACC
ntbR-rev	TGGCTCTCGCATTCAATCA
pelA-fwd	CTTCAGCCATCCGTTCTTCT
pelA- rev	GGCCATCTTGTAGCCATACT
cupA-fwd	ATGACCAGAACTTCGAACCC
cupA- rev	TCGCCGCTGAATGTGATAG
fleS-fwd	GGATGACGGTCACGAGATTT
fleS-rev	GTCGTTGAGCAGGATCAGTT
fleR-fwd	CAAGCCGATCAACCTGGATA
fleR-rev	ACCGACAGGCGATAGTAGA
fleQ-fwd	CTACTACCGCCTCAACGTATTC
fleQ-rev	GCTTCTCGTGCTCCATCC
flgK-fwd	CAATACCCTCTACGACCAACTG
flgK-rev	CTGGCTGAGATTGTTCACCT
fliC-fwd	CGACCAACTGAACTCCAACT
fliC-rev	GTGATGGTCAGTACACCC TTG
fliD-fwd	GAAACTGACCACCCAGTTCA
fliD-rev	AGGTCTGGTCGGTCACTT
flgD-fwd	GGTGGCCCAGTTGAAGAA
flgD-rev	CGACTGGTAGTTGGAGAGAATG
flgE-fwd	GAGAACAGCTGGACCATGAA
flgE-rev	CCGTCGATCAGCACTTTCA

The restriction endonuclease sites are underlined.

8. REFERENCES

Liberati, N.T., Urbach, J.M., Miyata, S., Lee, D.G., Drenkard, E., Wu, G., Villanueva, J., Wei, T., and Ausubel, F.M. (2006). An ordered, nonredundant library of *Pseudomonas aeruginosa* strain PA14 transposon insertion mutants. *Proc. Natl Acad Sci* 103**,** 2833-2838.

Schweizer, H. (1991). *Escherichia-Pseudomonas* shuttle vectors derived from pUC18/19. *Gene* 97**,** 109-112.
